# Whole-Genome Sequencing of Three Clonal Clinical Isolates of *B*. *cenocepacia* from a Patient with Cystic Fibrosis

**DOI:** 10.1371/journal.pone.0143472

**Published:** 2015-11-24

**Authors:** Ruth R. Miller, Trevor J. Hird, Patrick Tang, James E. A. Zlosnik

**Affiliations:** 1 School of Population and Public Health, University of British Columbia, Vancouver, British Columbia, Canada; 2 British Columbia Centre for Disease Control, University of British Columbia, Canada, Vancouver, British Columbia, Canada; 3 Centre for Understanding and Preventing Infection in Children, Department of Pediatrics, Faculty of Medicine, University of British Columbia, Vancouver, British Columbia, Canada; 4 Department of Pathology and Laboratory Medicine, Faculty of Medicine, University of British Columbia, Vancouver, British Columbia, Canada; ENEA Casaccia Research Centre, ITALY

## Abstract

*Burkholderia cepacia* complex bacteria are amongst the most feared of pathogens in cystic fibrosis (CF). The BCC comprises at least 20 distinct species that can cause chronic and unpredictable lung infections in CF. Historically the species *B*. *cenocepacia* has been the most prevalent in CF infections and has been associated in some centers with high rates of mortality. Modeling chronic infection by *B*. *cenocepacia* in the laboratory is challenging and no models exist which effectively recapitulate CF disease caused by BCC bacteria. Therefore our understanding of factors that contribute towards the morbidity and mortality caused by this organism is limited. In this study we used whole-genome sequencing to examine the evolution of 3 clonal clinical isolates of *B*. *cenocepacia* from a patient with cystic fibrosis. The first isolate was from the beginning of infection, and the second two almost 10 years later during the final year of the patients’ life. These isolates also demonstrated phenotypic heterogeneity, with the first isolate displaying the mucoid phenotype (conferred by the overproduction of exopolysaccharide), while one of the later two was nonmucoid. In addition we also sequenced a nonmucoid derivative of the initial mucoid isolate, acquired in the laboratory by antibiotic pressure. Examination of sequence data revealed that the two late stage isolates shared 20 variant nucleotides in common compared to the early isolate. However, despite their isolation within 10 months of one another, there was also considerable variation between the late stage isolates, including 42 single nucleotide variants and three deletions. Additionally, no sequence differences were identified between the initial mucoid isolate and its laboratory acquired nonmucoid derivative, however transcript analysis indicated at least partial down regulation of genes involved in exopolysaccharide production. Our study examines the progression of *B*. *cenocepacia* throughout chronic infection, including establishment of sub-populations likely evolved from the original isolate, suggestive of parallel evolution. Additionally, the lack of sequence differences between two of the isolates with differing mucoid phenotypes suggests that other factors, such as gene regulation, come into play in establishing the mucoid phenotype.

## Introduction

Bacteria belonging to the *Burkholderia cepacia* complex (BCC) are highly problematic pathogens in people with cystic fibrosis (CF). There are at least 20 distinct species of bacteria in the BCC, and all except for *B*. *ubonensis* have been isolated from the lungs of people with CF [1–3]. Historically, *B*. *cenocepacia* has been considered the most problematic species. Epidemic spread of *B*. *cenocepacia* has previously resulted in high rates of prevalence of this species in CF populations [4–6]. Furthermore, *B*. *cenocepacia* has been associated with high rates of mortality in some centers as well as exhibiting an elevated risk for death following lung transplantation [7–12], and high levels of intrinsic resistance to antimicrobial agents [6,11,13–16].

Further complicating our understanding of the pathobiology of BCC is their unpredictable infection outcome. Some people will experience severe and rapidly fatal infections, while others will experience a mild infection with little impact on lung function [17,18]. Most seriously, in some instances BCC bacteria can result in a rapidly invasive and typically fatal necrotizing pneumonia known as ‘cepacia syndrome’ [19]. The causes of these disparate outcomes are not understood and there are limited studies on adaptation of BCC bacteria to chronic infection in the CF lung.

We have previously described the prevalence of the mucoid phenotype in clinical sequential clonal isolates of BCC bacteria [20]. Both mucoid and nonmucoid BCC can cause infection in the CF lung, however during chronic infection phenotype switches are predominantly from the mucoid to nonmucoid phenotype. Significantly we have also shown that the degree of mucoidy displayed by clinical isolates of BCC is inversely related to both subsequent lung function decline and survival [17]. Supporting the notion that nonmucoid BCC are more virulent in the CF lung, proteomic and transcriptomic studies on a mucoid/nonmucoid clonal pair of isolates of *B*. *cenocepacia* from the late stage of a CF infection revealed elevated production of putative virulence determinants in the nonmucoid isolate [21]. Additionally, it was previously found that the nonmucoid variant formed biofilm and produced quorum sensing signaling molecules, while the mucoid variant demonstrated lower interaction with human leukocytes [22].

The genetic determinants of the mucoid phenotype are unknown, however, various genes have been hypothesized to be involved in exopolysaccharide (EPS) production. *bce-I* and *bce-II* are the only genes that have been shown experimentally to be involved in EPS production in *Burkholderia* [23]. Underlining the complexity of EPS production, BCC are thought to be able to produce at least seven different EPS [24], of which the isolates used in this study have been shown to produce at least three [22]. Additional EPS producing clusters that have been putatively identified include the *wcb* cluster, BCAM1330-BCAM1340 [25], as well as others (see Table 3 in: [26])

Next-generation whole-genome sequencing (WGS) now allows high-resolution insight into genomic changes that occur between sequential clonal isolates taken from patients at different time points during infection. This technology has already been employed to examine longitudinal isolates from a large outbreak of *Burkholderia dolosa* in CF patients in Boston [27]. In this study the authors examined sequential isolates from 14 patients and found evidence of 17 genes that were under strong selective pressure during chronic infection. Notably, the mutations they defined included gain of function mutations in antibiotic resistance and O-antigen switching. The only WGS data to date that has examined adaptation of *B*. *cenocepacia* to chronicity is a study examining experimental evolution of 6 populations across 1,050 generations in a biofilm model of evolution [28]. These data have shown, like the *B*. *dolosa* study, that there is selection of a limited number of pathways during this model of chronic infection and indeed this study also identified genes involved in LPS modification.

In this study we have used WGS to investigate: i) the evolution of *B*. *cenocepacia* during a well characterized single case of infection by comparing the initial isolate with two other isolates taken from later during infection and ii) the genetic similarities between exopolysaccharide production variants taken from chronic infection and derived *in-vitro* following antibiotic pressure of the original mucoid isolate from this infection.

## Methods

### Isolates

The isolates we investigated in this study have been described previously [17,22]. C3921, C8963 and C9343 are sequential isolates from the same patient. C3921-CTZ32G is an *in-vitro* derived nonmucoid isolate of the mucoid C3921 following exposure to super-MIC levels of ceftazidime (chosen because of its use in treating CF lung infections, including this patient’s BCC infection) [17]. Isolates were stored at -80°C in Mueller Hinton broth supplemented with 8% DMSO and have been minimally passaged since their original isolation.

### DNA extraction

Bacteria were revived from freezer stocks on Columbia Blood Agar plates at 37°C and single colony purified. Overnight cultures were established by inoculating several discrete colonies from the purified plate into 3 ml of Luria Bertani broth (10 g l^-1^ tryptone, 5 g l^-1^ yeast extract and 1- g l^-1^ sodium chloride) in a 15 ml snap top tube and incubating overnight at 37°C on an orbital rotating platform. DNA extraction was then performed exactly as per the Gram-negative bacteria protocol from a Qiagen Gentra Puregene Kit.

### RNA extraction

RNA was extracted from 16-hour cultures (OD_600_ 1.2) grown in yeast extract mannitol broth (4 g l ^-1^ mannitol and 0.5 g l ^-1^ yeast extract) using the Qiagen RNeasy Mini Kit. RNA was eluted in water and genomic DNA was removed using Turbo DNAse (Life Technologies) and stored at -80°C until use.

### Quantitative RT-PCR

cDNA synthesized from 10ng of extracted RNA using qScript DNA SuperMix (Quanta BioSciences) and 1:10 aliquots were used for subsequent quantitative PCRs. Primers for transcripts *bce-B*, *bce-E*, *bce-G*, *bce-R*, encoded by the cepacian biosynthesis genes, were used as previously designed by Ferreira *et al*., as were primers for the reference gene *gyrB* [29]. Reactions were assembled in a final volume of 25 μl containing 12.5 μl of 2X SYBR Green, 10.5 μl nuclease-free H_2_O, 1 μl cDNA and 1 μl of a 5 μM stock of primer pairs. Negative controls of nuclease-free H_2_O and RNA that had not undergone cDNA synthesis were used. Reactions were performed using a ViiA 7 Real-Time PCR System (Life Technologies). An initial cycle of 50°C for 2 mins was followed by 95°C for 2 mins for denaturation. There were then 40 cycles of 95°C for 15 secs followed by 57°C for 1 min followed by a melting curve stage. The ΔΔC_*T*_ was used to calculated relative gene expression by normalizing all transcripts to the *gyrB* reference. All experiments were performed in triplicate using three separate biological samples and standard errors of the mean calculated.

### DNA sequencing

Indexed sequencing libraries were created using the Nextera kit (Illumina, San Diego, CA) according to the manufacturer’s instructions. The libraries were sequenced on either the Illumina MiSeq to generate paired-end 2x250bp reads or Illumina HiSeq2500 to generate paired-end 2x150bp reads at the McGill University and Genome Quebec Innovation Center, Montreal, Canada. All four sequences have been deposited in the European Nucleotide Archive at the European Bioinformatics Institute under the study accession number PRJEB9630 (http://www.ebi.ac.uk/ena/data/view/PRJEB9630).

### Bioinformatics

Illumina adapters were removed from raw fastq files using cutadapt [30], after which reference based mapping to the *Burkholderia* J2315 reference [26] was performed, separately for each sample, using bowtie2 [31] with options:—phred33—local—dovetail—maxins 850. Variant positions were called using samtools [32], and to ensure high confidence, were filtered based on five parameters: (i) minimum read depth of five with at least one read in each of the forward and reverse direction; (ii) maximum depth not greater than the highest 2.5% of the distribution for the sample; (iii) minimum root-mean-square read mapping quality of 30; (iv) minimum of 75% of reads supporting the consensus call; (v) calls required to be homozygous under the diploid model assumed by samtools; using a bespoke Python script.

*De novo* assembly was performed on the unmapped reads following assembly with bowtie2, as well as the entire read set, using Velvet [33] in conjunction with the VelvetOptimiser (http://bioinformatics.net.au/software.velvetoptimiser.shtml). All contigs >500bp (mean 200 and 1412 per sample for unmapped and all reads respectively) were used in downstream analyses. To identify large regions varying between samples and not present in J2315, contigs produced from the unmapped reads were entered into Mauve [34] and NUCmer [35] to identify unique regions. Confirmation of any regions identified was then performed using BLAST searches of the region against the Velvet contigs made from the entire read set, and if the region was not present in every sample it was deemed a variant. Larger variants between samples and present in the reference, were identified using a window based approach on the reference based assembly, where any region of 1kb with >500bp different between two samples was identified. BLAST Ring Image Generator (BRIG) was used to generate visual genome comparisons with the J2315 reference [36] with low-confidence calls in the query being assigned the corresponding base from the reference sequence.

### Investigation of selected regions

Five regions implicated in EPS biosynthesis: *bce-I* (BCAM0854-BCAM0864), *bce-II* (BCAM1003-1011), *wcb* (BCAL3218-3245), BCAM1330-1349 [25] and the BCESM region were examined by extracting their sequence from the J2315 reference and using it as a BLAST query against the *de novo* contigs > 1kb produced from the entire read set. The regions from the contigs that matched the query were then aligned and compared. Since BCESM is a longer region, its investigation required manual contig joining.

## Results

**Fig 1:**
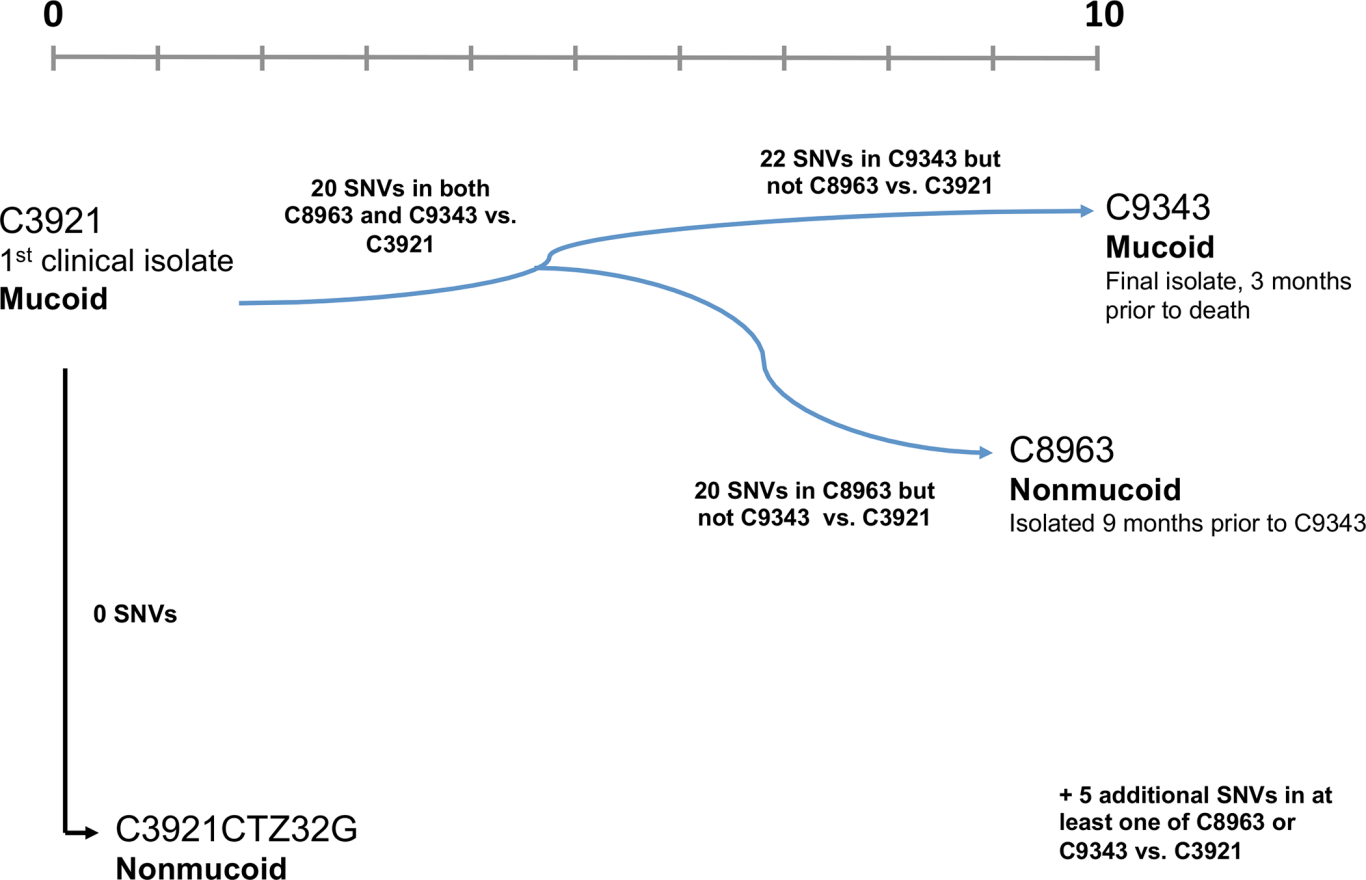
Isolates examined in this study. Four isolates were sequenced in this study: i) C3921 –the first isolate of *B*. *cenocepacia* from this patient, this isolate displays the mucoid phenotype; ii) C8963 –isolated 9 years and 3 months after the initial isolate, this isolate displays the nonmucoid phenotype; iii) C9343 –isolated 10 years after C3921 and 3 months prior to the death of this patient, this isolate was mucoid and iv) C3921-CTZ32G a nonmucoid variant of C3921 isolated in the laboratory following exposure to higher than MIC levels of the antibiotic ceftazidime [17]. Scale is time in years from first isolate.

In order to gain insight into changes in *B*. *cenocepacia* bacteria during chronic infection we sequenced three clinical isolates from a lung infection in a person with cystic fibrosis, as well as one laboratory derived nonmucoid variant (Fig 1). C3921 is the first stored isolate from this infection and displays the mucoid phenotype. C8963 was isolated 9 years and 3 months after C3921, while C9343 was isolated 10 years after C3921 and is also the final isolate we have stored for this patient.

**Fig 2:**
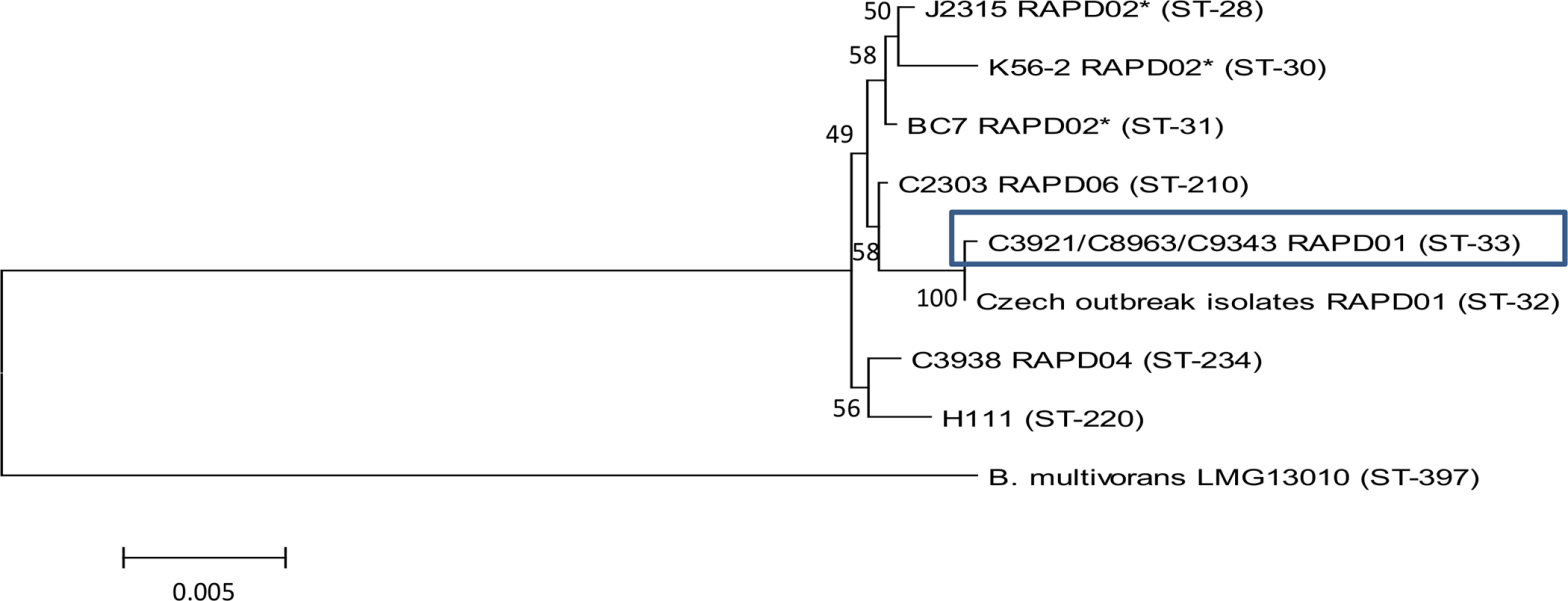
Phylogenetic analysis of the concatenated MLST alleles for isolates of *B*. *cenocepacia recA* subgroup A representing common epidemic clones or isolates commonly used in laboratory studies of this species. A neighbor-joining tree was constructed using the Jukes-Cantor method for computing evolutionary distances. The branch lengths, indicated by the scale bar, are measured as the number of substitutions per site. *B*. *multivorans* type strain, LMG13010, was used as an outlier. Bootstrap values (from 1000 replicates) are shown next to the branches. * = isolates belonging to the ET-12 lineage. The tree was constructed using MEGA6 [38].

The whole-genome sequence data at the 7 alleles used for MLST in BCC showed that all isolates were ST-33. Comparison of concatenated MLST gene fragments from our isolates to *B*. *cenocepacia* strains representative of the clonal diversity in the *recA* subgroup of this species (Fig 2), showed that ST-33 is most closely related to ST-32, which is a trans-continental epidemic strain that has been responsible for infections in Canadian patients and a large outbreak of *B*. *cenocepacia* in CF population in the Czech Republic [4,37].

### Overview of Whole-Genome Sequencing Results

We chose to map our results against the completed genome sequence for *B*. *cenocepacia* J2315 because J2315 is the type strain for the *B*. *cenocepacia* species, belongs to the same *recA* group A evolutionary lineage as the isolates used in our study and because the J2315 genome is complete, very well annotated and actively curated at www.burkholderia.com [26,39]. A mean of 1.2 million reads were produced per sample, of which a mean of 85.5% (range 83.9–86.7%) were mapped to the J2315 reference, resulting in a mean coverage of 89.4% of the reference with median depth of 57. After filtering, calls were made for a median of 81.4% of the reference.

In order to identify any regions in our samples that may not be present in the J2315 reference, *de novo* assembly was performed on all reads that did not map to J2315. This resulted in 84 contigs. 56 contigs were excluded as either common conserved regions between many bacteria, or contamination if they mapped to species other than *Burkholderia* that are considered unlikely to undergo horizontal transfer with *Burkholderia* (*Herminiimonas*, *Laribacter*, *Acidovorax*, *Homo sapiens*). 21/28 remaining contigs could be mapped to 7 different regions, each missing in one isolate. However, all of these contigs were <1200 bp and may have represented repetitive regions that may have been difficult to map. Interestingly 6/7 of the missing regions were in C9343, and one was in C8963.

**Fig 3:**
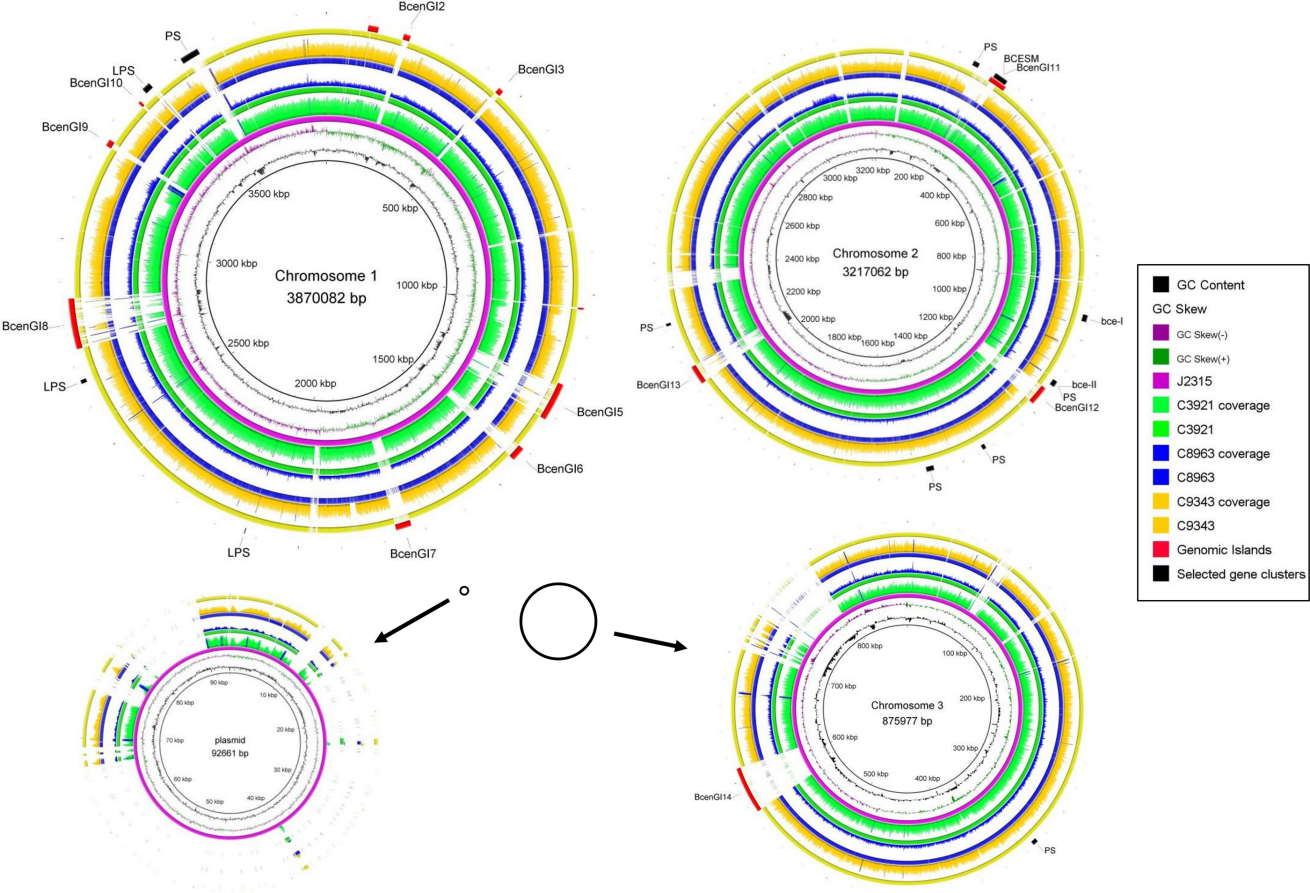
Genome sequences of isolates of *Burkholderia cenocepacia* C3921 (green), C8963 (blue) and C9343 (gold) compared to the reference genome of *B*. *cenocepacia* J2315 (purple), created using BRIG. Chromosomes 1 and 2 are drawn to scale. Black circles depict the relative sizes of chromosome 3 and the plasmid. To avoid implying absence where there was a lack of significance in the sequence, all low-confidence query sequences were called as their corresponding base pair in the J2315 sequence. Sequence coverage depth drawn with a maximum value cutoff of 150. The outer rings show the location of genomic islands described in J2315 [26]. PS = putative or known polysaccharide synthesis genes. LPS = lipopolysaccharide biosynthesis genes. BCESM = *Burkholderia cepacia* epidemic strain marker.

Comparative analysis of the assembled sequences using BRIG indicated that, compared to J2315, the RAPD01/ST-33 clonal lineage represented by these isolates lacks several regions (Fig 3). Notably, several genomic islands appear to be absent (BcenGI2, BCenGI3 and BcenGI9). It is also notable that several other genomic islands have poor coverage in our sequence results and it is not clear whether these are present or not in the lineage of *B*. *cenocepacia* represented by these sequences. Additionally, a region on chromosome 1 containing the *wcb* genes thought to encode a capsular polysaccharide homologous to a capsular polysaccharide characterized in *B*. *pseudomallei* K96243, was missing from all the isolates we sequenced.

In total we confidently identified 76 single nucleotide variants (SNVs) between our isolates and the J2315 reference genome (Table 1). Of these, 15/76 (20%) were in non-coding regions. Of the 61 SNVs identified in genes, 43/61 (70%) coded for non-synonymous mutations and 18/61 (30%) coded for synonymous mutations. 51 genes contained a single SNV while 5 genes contained 2 SNVs.

**Table 1:** Summary of SNVs.

Position in J2315*	Qualifier	Gene Name	Gene Product	J2315	C3921	C8963	C9343	Amino acid change	SNV effect	Predicted functional effect	Score**	Transcriptome^+^	Proteome^+^
**SNVs in Both C8963 and C9343 compared to C3921**													
263702	BCAL0227	*rpoC*	DNA-directed RNA polymerase subunit beta	C	C	T	T	S109L	NS	Deleterious	-5.06		
359602	BCAL0331		putative stringent starvation protein A	C	C	T	T	R162C	NS	Deleterious	-7.97		
880552	BCAL0809		HPr kinase/phosphorylase	G	G	A	A	T226I	NS	Deleterious	-5.35		
1767570	BCAL1609		binding-protein-dependent transport system inner membrane protein	C	C	T	T	G17D	NS	Deleterious	-5.40		
1874411	BCAL1700	*orbA*	ornibactin receptor	G	G	A	A	G598D	NS	Neutral	0.96		
2425667	BCAL2198	*iscS*	cysteine desulfurase	G	G	A	A	P338L	NS	Deleterious	-9.51		1.61
3759013	BCAL3430	*ampD*	N-acetyl-anhydromuranmyl-L-alanine amidase	G	A	G	G	A82V	NS	Deleterious	-3.85		
3764522	BCAL3436	*proS*	prolyl-tRNA synthetase	G	G	A	A		S				
4879783	Non-coding			A	A	G	G		-				
5360319	BCAM1342		putative sigma-54 interacting transcriptional regulator	T	T	C	C	F164L	NS	Deleterious	-5.16		
5385281	BCAM1362		putative penicillin-binding protein	G	A	G	G		S				
5438246	BCAM1409		hypothetical protein	C	C	T	T		S				
5791984	BCAM1722		LysR family regulatory protein	A	A	G	G	C242R	NS	Neutral	-1.09		
5791985	BCAM1722		LysR family regulatory protein	C	C	G	G		S				
6304869	BCAM2177		hypothetical protein	G	G	A	A	R16K	NS	Neutral	-1.35		
6577710	Non-coding			C	C	T	T		-				
6651209	BCAM2461		putative inosine-uridine preferring nucleoside hydrolase	T	T	G	G	D289A	NS	Neutral	-0.50		
7095726	BCAS0007		TetR family regulatory protein	G	T	G	G	K47N	NS	Deleterious	-4.96		
7423790	BCAS0302		hypothetical protein	G	G	T	T	C249F	NS	Deleterious	-7.27		
7625727	BCAS0471		outer membrane efflux protein	C	C	T	T		S				
**SNVs only in C8963 versus C3921**													
35492	BCAL0032	*atpF*	F0F1 ATP synthase subunit B	G	G	A	G		S				
381576	BCAL0351		putative type VI secretion system protein TssM	C	C	T	C		S				
548717	Non-coding			C	C	T	C		-				
1123579	BCAL1039		ABC transporter ATP-binding membrane protein	A	A	G	A	V175A	NS	Deleterious	-3.92		
1473608	BCAL1345		putative TonB-dependent siderophore receptor	C	C	T	C	A96V	NS	Deleterious	-3.84		
1601953	BCAL1448	*valS*	valyl-tRNA synthetase	G	G	C	G	T14S	NS	Neutral	0.75		
2115945	BCAL1917		hypothetical protein	C	C	T	C		S				
2544053	BCAL2292		putative bipolymer transport protein	C	C	T	C	A7V	NS	Neutral	-2.01	0.47	
2603160	Non-coding			G	G	A	G		-				
3580361	BCAL3271		thioredoxin	C	C	T	C		S				
3616073	BCAL3301	*oxyR*	oxidative stress regulatory protein	T	T	C	T	H203R	NS	Neutral	0.12		
3616430	BCAL3301	*oxyR*	oxidative stress regulatory protein	T	T	C	T	Q84R	NS	Neutral	-1.29		
3782820	BCAL3453	*secA*	preprotein translocase subunit SecA	A	A	G	A	V207A	NS	Deleterious	-3.80		
3934083	BCAM0059	*pcaJ*	3-oxoadipate CoA-transferase subunit B	C	C	T	C	R6C	NS	Deleterious	-6.62		
5445821	BCAM1417		two-component regulatory system sensor kinase	G	G	T	G	L28M	NS	Neutral	-1.69	0.43	
5825895	BCAM1745		putative magnesium-transporting ATPase	G	G	A	G	A661T	NS	Deleterious	-3.94		
5921203	BCAM1831		putative cyclase	G	G	A	G	A38T	NS	Deleterious	-2.54		
6286440	BCAM2168		putative amylo-alpha-1,6-glucosidase	G	G	A	G		S				
6837958	Non-coding			A	A	T	A		-				
7202485	Non-coding			C	C	T	C		-				
**SNVs only in C9343 versus C3921**													
405727	BCAL0374	*prfA*	peptide chain release factor 1	C	C	C	T	E161K	NS	Deleterious	-4.00		
864111	Non-coding			T	T	T	C		-				
1894555	Non-coding			T	T	T	C		-				
2374985	BCAL2149		HhH-GPD superfamily base excision DNA repair protein	T	T	T	C	E164G	NS	Deleterious	-5.61		
2423968	BCAL2195	*hscB*	co-chaperone HscB	T	T	T	C		S				
2717069	BCAL2452		LysR family regulatory protein	T	T	T	C	L112P	NS	Deleterious	-6.18	18	
2871548	Non-coding			G	G	G	T		-				
2873956	BCAL2615		putative exported outer membrane porin protein	G	G	G	A		S				
3037135	BCAL2765	*rpsT*	30S ribosomal protein S20	C	C	C	T		S				
3043868	BCAL2772		putative AMP-binding enzyme	T	T	T	G	V494G	NS	Neutral	-1.75		
3201111	BCAL2920		subfamily M48A metalopeptidase	T	T	T	C	W185R	NS	Deleterious	-12.9		
3319069	BCAL3029		putative alkane monooxygenase	T	T	T	C	F144L	NS	Neutral	-1.60		
3601711	BCAL3287		putative FAD-binding oxidase	G	G	G	A		S				
3605061	BCAL3289	*glcE*	glycolate oxidase FAD binding subunit	G	G	G	A	G257S	NS	Neutral	0.10		
4114326	BCAM0207		putative tyrosine-protein kinase	C	C	C	T		S			62.1	
4375146	BCAM0451		putative extracellular endonuclease/exonuclease/phosphatase family protein	C	C	C	G	P39A	NS	Neutral	-0.28		
4807037	BCAM0851		hypothetical protein	C	C	C	T		S			0.31	
5546631	Non-coding			T	T	T	A		-				
6104972	BCAM2017		ABC transporter ATP-binding protein	G	G	G	A		S				
6117131	BCAM2027a		hypothetical protein	G	G	G	A	S53L	NS	Deleterious	-3.05		
6283639	BCAM2165	*penA*	putative beta-lactamase	T	T	T	C	N155S	NS	Deleterious	-4.99		
7405450	BCAS0284		Major Facilitator Superfamily protein	C	C	C	T	R70W	NS	Deleterious	-5.66		
**SNVs in at least one of C8963 and C9343 compared to C3921**													
1839129	BCAL1679		putative fimbrial chaperone	C	C	A	N	N175K	NS	Deleterious	-6.00		
1870872	BCAL1698	*orbK*	ornibactin biosynthesis protein	G	G	C	-	W241C	NS	Deleterious	-13.0		
3863278	BCAL3527	*gspD*	type II secretion system protein D	T	T	A	N	Q502L	NS	Deleterious	-3.44		
5197275	Non-coding			C	C	N	T		-				
6283537	BCAM2165	*penA*	putative beta-lactamase	T	T	C	N	E189G	NS	Deleterious	-5.61		
**Additional SNVs in C8963 and C9343 compared to J2315**													
6844295	Non-coding	* *		T	N	T	G		-				
3238151	BCAL2957	*gyrA*	DNA gyrase subunit A	G	N	A	A	T83I	NS	Deleterious	-4.35		
4392099	BCAM0467	* *	hypothetical protein	A	N	A	C	K18Q	NS	Deleterious	-3.73		
4392100	BCAM0467	* *	hypothetical protein	A	N	A	G	K18R	NS	Neutral	-2.46		
4393598	BCAM0468	* *	hypothetical protein	A	N	A	G	F339W	NS	Neutral	-1.88		
4393599	BCAM0468	* *	hypothetical protein	G	N	G	A		S				
4393601	Non-coding	* *		C	N	C	A		-				
4393634	Non-coding	* *		A	N	A	C		-				
4393637	Non-coding	* *		C	N	C	T		-				

* = position relates to the full concatenated genome of all three chromosomes of the sequenced J2315 *B*. *cenocepacia* genome.

** = Predicted functional effect and score calculated by Provean (http://provean.jcvi.org/index.php). A value of -2.5 or lower was used to determine if a mutation was deleterious or neutral.

^+^ = Previous transcriptome and proteome data comparing C8963 and C9343 [21]. Values are relative overexpression in the mucoid C9343 vs the nonmucoid C8963.

N = insufficient evidence to make a confident base call at this position.

### Identification of SNVs In Clinical Isolates Following Evolution in the CF Lung

Compared to C3921, C8963 has 40 SNVs of which 34 were in coding regions. 24/34 SNVs in coding regions of C8963 were non-synonymous and 15 of these are predicted by *in-silico* analysis to a have deleterious effect on the encoded protein. Our data also revealed there were 42 SNVs in C9343 compared to the initial isolate C3921. Of these 42 SNVs, 36 were identified in coding regions of which 24 are predicted to code for non-synonymous mutations and 16 of these predicted to be deleterious mutations (Table 1 and Fig 1). These SNVs were categorized by function, which revealed the most commonly mutated genes were those involved in genetic regulation and metabolism both before and after the divergence of C8963 and C9343 (9 SNVs in both categories), while C8963 also acquired a number of SNVs in secretion and transport independent of C9343 (N = 3) (Table 2).

**Table 2:** Functional categorization of non-synonymous SNVs.

Function*	Both C8963 and C9343	C8963 only	C9343 only
Genetic regulation	4	3	2
Metabolism	4	2	3
Iron acquisition	1	1	0
Outermembrane	1	0	0
Secretion/Transport	0	3	1
Antibiotic resistance	0	0	1
Unknown	1	1	2
Other	2	1	2

* = Genes were grouped into functional categories based on their COG annotation in the Burkholderia.com database [39]. SNVs included in this analysis were only those for which there was a confident base-pair call in all three isolates (C3921, C8963 and C9343). In addition to these SNVs, 4 others were detected in C8963 for which there was not a confident base-pair call in C9343, these were 2 genes involved in secretion (BCAL1679 and BCAL3527/*gspD*); one gene involved in iron acquisition (BCAL1698/*orbK*); and one gene involved in antibiotic resistance (BCAM2615/*penA*). There were also 4 genes with SNVs in C8963 and C9343 relative to J2315 for which there weren’t confident base-pair call. These were 1 gene involved in antibiotic resistance (BCAL2957/*gyrA*) and, 3 SNVs in 2 genes of unknown function (2 in BCAM0467 and 1 in BCAM0468)

Based on the confidently identified SNVs, these data are suggestive of an average evolutionary rate of 5.3 x 10^−7^ SNVs/bp/year during the course of this CF infection. This rate is similar, albeit slightly higher, to that found in other studies of *Burkholderia* in CF infection, for example 3.28 x 10^−7^ SNVs/bp/year in *Burkholderia dolosa* [27]. Notably, C8963 and C9343 did not share all SNVs in common. We observed 20 SNVs in common that were present in both C8963 and C9343 compared to the initial isolate from this patient, and 20 and 22 SNVs that were unique to C8963 and C9343 respectively (Fig 1 and Table 1). Based on the average SNV rate these data suggest that C8963 and C9343 may have undergone significant parallel evolution, having diverged after approximately four and half years in the CF lung.

There were also an additional number of SNVs or putative SNVs identified. Specifically, there were 5 in either C8963 or C9343 for which there was not a confident identification in the other late-stage isolate to assign them common to both C8963 and C9343 but not C3921. Finally there were 9 instances of SNVs where C9343 and or C8963 differed from the J2315 reference sequence, but were too low confidence in C3921. Among these was a SNV in *gyrA*, which has previously been identified as commonly mutated in *B*. *dolosa* during chronic infection [27].

C9343 has previously been shown to have a number of alterations in its pulsed-field gel electrophoresis pattern compared to C8963 following *Xba*I digestion of genomic DNA [22]. Investigation of larger deletions with respect to the reference revealed three regions where C9343 had a deletion compared to the other samples, as well as the reference. The first was a ~1600bp region on chromosome 1 corresponding to the genes *orbI*, *orbJ*, *orbK* and *pvdA* and the second a ~600bp region corresponding to a family M14 peptidase. Previous PCR and transcriptomic data has suggested that C9343 also has a substantial deletion in the *Burkholderia cepacia* epidemic strain marker (BCESM) [21]. The BCESM region contains a number of putative virulence determinants, including a quorum sensing signaling regulator. The deletion in C9343 can be seen in Fig 3 and is approximately 6 kb ranging from BCAM0211 to BCAM0256 in the J2315 reference.

### Mucoid C3921 and nonmucoid C3921-CTZ32G

Examination of the data from C3921 and the *in-vitro* derived nonmucoid isolate C3921-CTZ32G showed there were no confidently identified SNVs or other genomic changes in the sequence data between them. Because we had chosen to assemble our sequences against the somewhat distantly related J2315 reference genome, we also constructed a bespoke reference based upon the sample reads using Medusa [40]. However, we were also unable to identify any differences between C3921 and C3921-CTZ32G using this approach. Furthermore, we were unable to confidently identify any differences between these isolates by reducing the filtering criteria to a minimum depth of 2, minimum root-mean-square read mapping quality of 10, and minimum of 60% of reads supporting the consensus call (data not shown).

**Fig 4:**
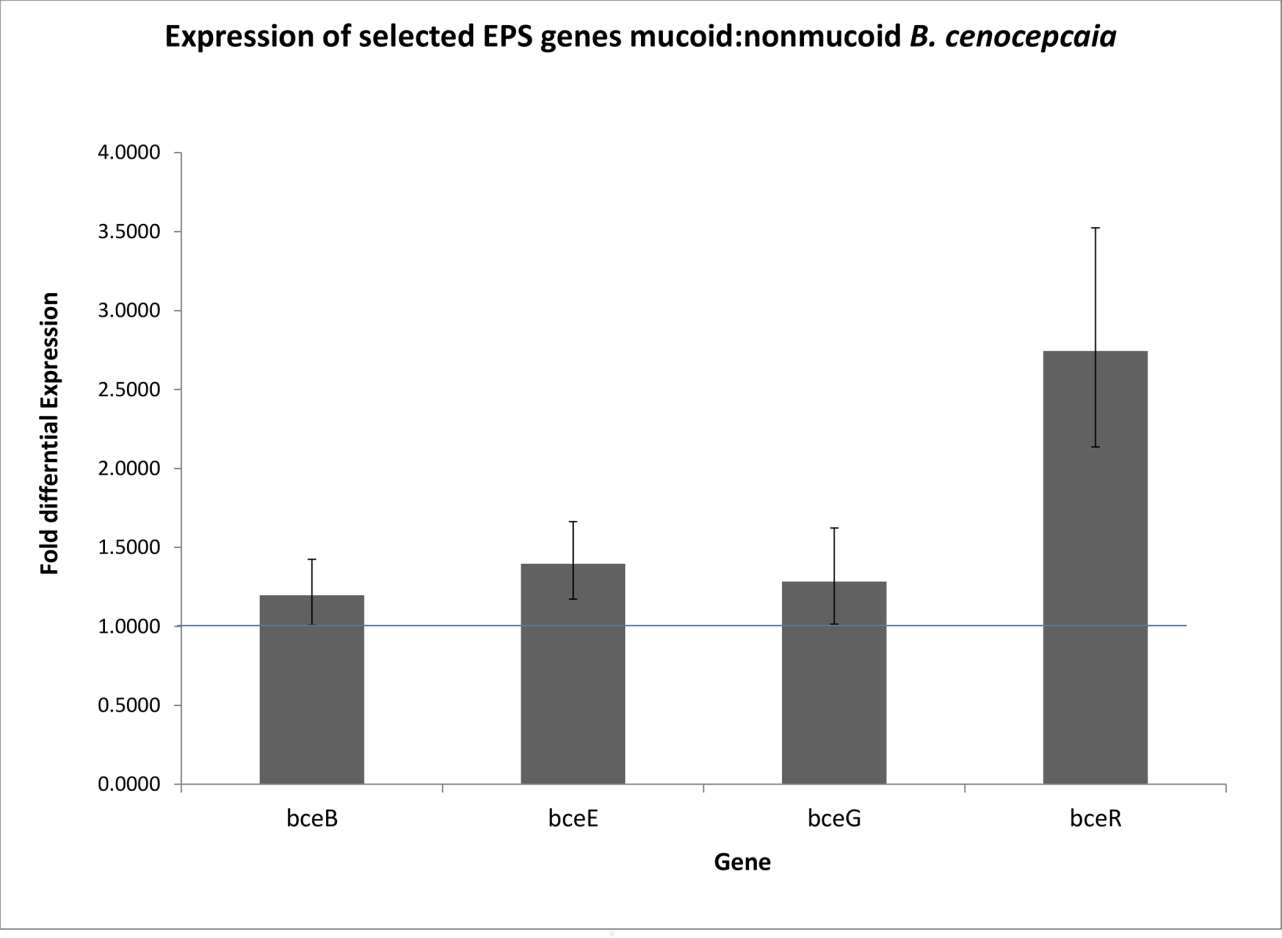
Quantitative RT-PCR of transcripts encoded by selected genes from the cepacian biosynthesis cluster show upregulation of *bce* transcripts in mucoid C3921 relative to its corresponding nonmucoid derivative C3921-CTZ32G. RNA extracted from triplicate stationary phase cultures of bacteria (16 hour) grown in yeast extract media was assayed by quantitative RT-PCR in triplicate, using transcripts from the *gyrB* gene to normalize expression values between experiments. Results are expressed relative to the nonmucoid isolate, C3921-CTZ32G, and error bars represent standard error of the mean.

To determine if differential expression of genes involved in cepacian biosynthesis is associated with the mucoid phenotype in this strain, we examined data from a study that we have previously published comparing mucoid C9343 with nonmucoid C8963 at the transcriptomic level using a microarray. These data revealed there is differential expression of at least some of the genes involved in the cepacian biosynthesis. *bce-F* (BCAM0859), *bce-G* (BCAM0860) and *bce-R* (BCAM1008) were overexpressed in mucoid C9343 2.9, 4.0 and 2.1 fold respectively [21]. Because there are numerous genetic differences between mucoid C9343 and nonmucoid C8963, we sought to examine transcription of the cepacian biosynthetic operon in C3921 and C3921-CTZ32G. Consistent with our previous data, quantitative RT-PCR of select genes from the *bce-I* and *bce-II* clusters showed elevated expression of genes from both clusters in the mucoid isolate (Fig 4).

## Discussion

One of the more challenging aspects of the infections caused by BCC bacteria is their chronic nature, with infections lasting years, and sometimes decades, making *in-vitro* modeling of virulence challenging. Whole-genome sequencing offers the opportunity to understand adaptation at the genomic level ‘*in-vivo*’ by examining isolates taken at different stages of chronic infection. The data presented here are one of the first comparative analyses of *B*. *cenocepacia* longitudinal clinical isolates taken from a chronic cystic fibrosis infection. The aim of this study was to characterize at the genomic level three sequential clonal isolates of *B*. *cenocepacia* taken from the beginning (C3921) and end (C8963 and C9343) of a single and ultimately fatal cystic fibrosis infection (Fig 1). These isolates are of interest because: i) they were part of an epidemic clone responsible for a number of infections in the Vancouver CF population [41]; ii) they are closely related by MLST (differing by a single base pair) to an epidemic clone responsible for a large outbreak in the Czech CF population, where there was significant mortality due to cepacia syndrome [37,42] and iii) they display colony morphology differences, with the initial isolate (C3921) being mucoid while the later isolate (C8963) is nonmucoid and the last isolate (C9343) is mucoid [21,22].

Our data reveal differences between the epidemic lineage represented by the isolates sequenced in this study and the more commonly studied Electrophoretic Type (ET)-12 lineage represented by the J2315 reference sequence (Fig 3). Notably, these isolates are missing several genomic islands encoded in the J2315 genome (BcenGI2, BcenGI3 and BcenGI9) and also a region encoding a putative capsular polysaccharide (Fig 3). This later observation is important in the context of the mucoid phenotype displayed on exopolysaccharide promoting agar plates by these isolates, indicating that these genes are not required for mucoidy. While these isolates were taken from an ultimately fatal CF infection, it is noteworthy that this infection lasted 10 years in a female patient. We have recently published data that showed, in the same population, median survival for females with *B*. *cenocepacia* was just 2.28 years [41]. It has also been previously speculated that there may be differences in virulence between the ET-12 and ST-32 lineages, the later of which is closely related to these isolates [4]. Therefore, these regions of difference may represent targets worthy of further investigation for their contribution to heightened virulence in BCC.

Three previous studies have looked at whole-genome sequences of other BCC following evolution in either lung infections caused by an epidemic strain of *B*. *dolosa* [27,43] or in an *in-vitro* model of biofilm evolution in *B*. *cenocepacia* H111 [28]. Our data extends these observations to a single CF lung infection caused by an epidemic lineage of *B*. *cenocepacia*. There have also been studies examining changes in a closely related bacteria, *B*. *pseudomallei*, a primary human pathogen also capable of establishing chronic carriage over decades [44]. In common with these studies, we also found mutations in *rpoC* as well as a number of genes related to central metabolism, iron metabolism and control of transcription, reinforcing these common themes in adaptation to chronic infection in the CF lung (Table 2). Interestingly we also detected SNVs in *gyrA* in both C8963 and C9343 in amino acid position T83 relative to J2315. Mutations in *gyrA* can confer resistance to fluoroquinolone antibiotics, which appears to be a common adaptation to growth in the CF lung in both *Burkholderia* and *P*. *aeruginosa* infections [27,45]. The mutations that we found in common between C8963 and C9343 may indicate features important to initial pathoadaptation to the CF lung. In particular genes involved in genetic regulation (N = 4) and metabolism (N = 4 plus 1 involved in iron acquisition) received the highest numbers of non-synonymous SNVs within each category (Table 2). However, it is important to interpret these results carefully. As with the studies on *B*. *dolosa* [27,43], this infection is part of an outbreak of *B*. *cenocepacia* that occurred before effective infection control [41] and therefore it is likely that these isolates were acquired directly from another CF lung infection. Although we do not know for certain if this patient acquired their *B*. *cenocepacia* from another patient, it is possible that these isolates had already undergone pathoadaptation to the CF lung and any changes that we observed were specific to adaptation in our case. Nonetheless, the genes involved in genetic regulation (BCAL0227/*rpoC*, BCAM1342, BCAM1722 and BCAS0007), in particular, represent interesting targets for future work to understand factors involved in *B*. *cenocepacia* evolution in CF lung infections.

Our finding that isolates C8963 and C9343 only shared around 50% of SNVs in common, compared to the initial isolate C3921, implies that these two isolates diverged and co-existed in parallel for almost half of the infection. This finding is consistent with the diverse community model proposed by Lieberman and colleagues, following their sampling of patients infected with an epidemic strain of *B*. *dolosa* [43]. In this model, an initial infecting strain diverges over time in the CF lung with the establishment of a range of stable sub-populations. Similar findings have also been recently reported for *B*. *pseudomallei* [46] and *P*. *aeruginosa* [47]. Therefore, to fully understand BCC infections in the CF lung, it appears that increased sampling is merited.

There was very little overlap between the SNVs identified in this study and the genes and proteins that we have previously described as differentially regulated between C8963 and C9343 [17]. Noteworthy genes that did overlap include the two SNVs in *oxyR* found in C8963 (Table 1). *oxyR* is known in other bacteria to be involved in regulating genes in response to oxidative stress [48]. This includes the gene *ahpC* which we have shown is overexpressed in C8963, therefore it is conceivable that these SNVs are involved in overexpression of *ahpC* [21]. Another notable SNV is in the gene BCAM0207, annotated as a tyrosine-protein kinase and part of a putative polysaccharide cluster, which contained a SNV in C9343. We previously found this to be overexpressed 62.1 fold in C9343 versus C8963. BCAM0207 is an orthologue of a gene in the cepacian biosynthesis operon, *bce-F*. *bce*-*F* has been previously shown to be required for cepacian biosysnthesis [49], as well as for invasion of epithelial cells [50]. While it is not essential for the mucoid phenotype in these isolates (as isolates with both variants of the SNV are mucoid), it is conceivable that this gene is involved in the EPS profile of this isolate and/or the severity of infection in this patient and this merits further study. Finally, in this study we also observed the absence of a significant part of the BCESM in C9343 (Fig 3), this contains a quorum sensor regulator and its loss could in part explain the loss of some of the apparently quorum sensing related phenotypes we have previously observed in C9343.

It is notable that we were unable to detect any genomic differences between the mucoid C3921 isolate and its nonmucoid derivative C3921-CTZ32G. BCC bacteria are known to be capable of producing a number of different exopolysaccharides [22,25,51–53]. However, the main exopolysaccharide produced in most clinical strains of BCC is ‘cepacian’ which is produced under the control of genes in the *bce-I* and *bce-II* loci (annotated on the J2315 genome sequence BCAM0854-BCAM0864 and BCAM1003-1011 respectively) [29]. Our study was unable to find any differences in these regions and in sequences both up and down stream that could account for the difference in morphology. It is, however, clear from these data that another previously described putative exopolysaccharide cluster of genes, *wcb*, (annotated on the J2315 genome sequence as BCAL3218 to BCAL3245 [25]) are not essential for the mucoid phenotype as they were absent in all strains including those elaborating the mucoid phenotype. Both the results of the RT-PCR (Fig 4) and an analysis of our previous transcriptomic data, show that there is differential expression of at least some genes involved in the cepacian biosynthesis. These data suggest, therefore, that there is genetic control of at least these genes and this may contribute to the lack of exopolysaccharide product. Because our study was not powered to fully complete the genome sequences, it seems likely that the genetic control of these genes is from a region that we have not identified. It is has been shown that the third chromosome (now thought to be a plasmid) does not appear to encode a regulator of exopolysaccharide production in *B*. *cenocepacia* H111 [54]. Nonetheless, it remains conceivable that there could be strain specific factors involved in control of exopolysaccharide production and these remain elusive.

One of the limitations of this analysis has been the lack of a reference genome for this lineage of *B*. *cenocepacia* which forced us to compare our data against the completed genome sequence for *B*. *cenocepacia* J2315. As can be seen from the MLST phylogeny (Fig 2), there is some distance between J2315 and our isolates, which reduces the quality of alignment, including causing a larger percentage of reads to be unmapped. However, we took care to investigate the reads that did not map to J2315 intensively, and were unable to find differences in them between C3921 and C3921-CTZ32G. We used strict filtering criteria of mapping results to prevent identification of false positive variant positions, thus it is possible that additional variation would have been identified if less stringent criteria were used. However, no differences between C3921 and C3921-CTZ32G arose when the filtering criteria were relaxed. The development of additional well annotated reference sequences for lineages of *B*. *cenocepacia* would be useful for future studies examining larger numbers of isolates from chronic infections. However, we did also conduct this analysis using a bespoke reference, and were still unable to determine any differences between the nonmucoid C3921-CTZ32G and its mucoid parent. Advances in genome sequencing technologies, which enable longer reads, will in the future enable easier assembly of sequence data.

In conclusion, these data complement existing studies, have identified genes that may be important in the pathobiology of *B*. *cenocepacia* in the CF lung, and help clarify our previously published data regarding isolates C8963 and C9343. Specifically, these data support the model of divergent evolution in the CF lung and extend this observation to one of the most common BCC species, *B*. *cenocepacia*. Future studies will examine larger numbers of isolates from infection and these will help further illuminate the genetic strategies used by *B*. *cenocepacia* to establish and maintain chronic infection in the CF lung.
